# We Aren’t Just ‘Slack’ing Off: Utilizing a Digital Tool to Connect Emerging Women Leaders in Global Health

**DOI:** 10.5334/aogh.3759

**Published:** 2022-09-12

**Authors:** Jennifer L. Weinberg, Amena El-Harakeh, Sandra Kiplagat, Aisha Ahmed Abubakar, Sloka Iyengar, Agustina M. Marconi, Tanaz M. Vaghaiwalla, Anna Kalbarczyk, Meagan Harrison

**Affiliations:** 1 Department of Nursing and Department of Health Sciences, Monmouth University, West Long Branch, NJ, USA; 2 City University of New York (CUNY) Graduate School of Public Health and Health Policy, New York, USA; 3 Department of Epidemiology, Florida International University, Miami, FL, USA; 4 Department of Community Medicine, Ahmadu Bello University Zaria, Kaduna, Nigeria; 5 American Museum of Natural History, New York, USA; 6 University Health Services, University of Wisconsin-Madison, Madison, WI, USA; 7 Department of Surgery, University of Tennessee Graduate School of Medicine, Knoxville, TN, USA; 8 Department of International Health, Johns Hopkins Bloomberg School of Public Health, Baltimore, MD, USA; 9 Johns Hopkins Center for Global Health, Johns Hopkins University, Baltimore, MD, USA

**Keywords:** global health, women, leadership, network, Slack, mentorship

## Abstract

**Background:**

Investing in women leaders in global health catalyzes growth and positive outcomes for individuals and their communities, yet large gender disparities persist in leadership within the field due to several barriers. The use of digital tools facilitates cross-institutional and international collaborations to allow individuals or groups to create or share information, ideas, career interests, and other forms of expression via virtual communities. Digital tools can dramatically expand access to and the quantity and quality of opportunities for networking, mentoring, and collaboration to support women in their professional development.

**Objectives:**

The objective of this paper is to document tangible examples of positive experiences, connections, or collaborations resulting from connecting with other participants in a Slack network. We aimed to evaluate this network to understand how to better build, model, and scale advantageous digital networks of women leaders in global health moving forward.

**Methods:**

Semi-structured interviews were conducted virtually with seven members of the Slack network from Africa and North America who volunteered to share their experiences. Transcripts of six of these interviews were analyzed for key points using thematic analysis to derive short vignettes from each interview.

**Findings:**

The findings of this study indicate that Slack is a highly beneficial tool for women in global health to use for facilitating job searches, mentoring opportunities, promoting project collaborations, and proposing programming and outreach ideas in a remote environment. We found distinct recommendations for utilizing this digital networking tool in a way that best supports and engages women in global health. It is important to spread awareness and ensure visibility of the network to recruit and maintain members, design the network in a way that inspires internal motivation, encourage consistent and meaningful engagement, send weekly emails, and maintain accessibility for a global membership base.

**Conclusions:**

The Slack network provides an engaging digital tool that facilitates communication, opportunities, and growth among women in global health. Digital tools such as Slack can help to increase opportunities for participants from low- and-middle-income countries to engage in the same networking and leadership opportunities as individuals from high-income countries. It remains critical to continue to build, advance, and scale advantageous networks like Slack to promote equity and accessibility among women leaders in the global north and south into the post-pandemic world.

## Introduction/Background

Global health is an inherently multidisciplinary and cross-cultural field which benefits from collaboration and a global mindset. Women comprise roughly 70% of the global health workforce, yet large gender disparities persist in leadership within the field [1]. Investing in building women leaders in global health catalyzes growth and positive outcomes for the individual, institution, community, and society [2].

### Digital Tools for Collaboration, Networking, and Mentorship

Networking, mentoring, and collaboration have long been crucial components of professional development. Traditionally, this has been accomplished via such platforms as trainings and career services at local institutions, formal and informal networking and mentorship through participation in in-person groups such as alumni networks, presentations and gatherings at local and national conferences, and writings in journals and other media.

These types of in-person events present many barriers to access. In-person communication can be impractical for group members across various time zones. The cost, logistics such as obtaining visas and travel, and taking time away from work and home responsibilities can prohibit participation in in-person conferences and other events for many, especially those from low- and middle-income countries. Additionally, during public health crises such as the COVID-19 pandemic, more innovative digital solutions are needed to allow ongoing interaction even when in-person meeting is limited. The use of digital tools to facilitate cross-institutional and international collaborations has the potential to dramatically expand access to and the quantity and quality of such opportunities. Digital tools provide a platform for broader networking and collaboration, mentoring, and longitudinal professional development experiences.

Social media tools are interactive technologies that allow individuals or groups to create or share information, ideas, career interests, and other forms of expression via virtual communities and networks such as like Twitter™, Facebook™, Instagram™, Linkedin™, Whatsapp™, Slack™, blogging platforms, and similar tools [3]. These types of digitally-mediated platforms can enable short- and long-term connections amongst geographically dispersed women working in various aspects of global health. Both informal collaborative networks and formal virtual conferences, projects, and meetings can be successfully accomplished via digital platforms that can help to evolve, expand the reach of, and compliment more traditional in-person networking opportunities.

Digital tools are increasingly used in various aspects of the health industry to facilitate connection for work, education, public health, and social purposes. For example, major medical organizations utilize social media to rapidly disseminate consensus and expert opinion, including sharing guidelines, protocols, and standardized operating procedure, and many major medical and healthcare-related conferences have gone virtual and/or integrated digital content to reach a broader audience [4]. Digital messaging platforms like Whatsapp continue to be used for social communication as well as expanding to serve as a powerful repository for sharing information [4].

Social media and other digital platforms have also been suggested as tools to help expand access to targeted communication around opportunities to expand women’s opportunities in global health leadership, especially for those in low- and middle-income countries (LMICs) [5]. Similarly, crowdsourcing of researchers in LMICs have identified a variety of digital methods for enhancing research mentorship including social networking platforms like Facebook, messaging apps like Slack and WhatsApp, podcasts, and online support and networking platforms like DataCamp™ and Coursera™ [6].

These solutions have the potential to facilitate the expansion of skills and opportunities to enable more women to become leaders in global health while helping to decolonize global health practices. Digital solutions can not only replicate traditional platforms for networking, mentoring, and collaboration but also offer innovative strategies and pathways for connection in new and expanded ways.

### Slack

Slack (Slack Technologies, San Francisco, California) is a growing cloud-based digital workspace and information management platform with 10 million daily active users around the globe. Over half of Slack’s daily active users are outside of the United States in more than 150 countries [7]. More than 750,000 businesses across a wide variety of industries use Slack every day. The platform provides a digital medium for team messaging, file sharing, and video/voice calls and integrates with thousands of additional digital tools, like Google Drive™, Zoom™, and Salesforce™ [8]. Slack is broadly accessible via the web, a desktop application, or a mobile application and offers several levels of service, including a free basic option and upgraded tiers [9].

The design of Slack resembles a social media platform that creates a collaborative digital culture. This ‘enterprise social network’ (ESN) is a team-based platform that allows users to create a series of perpetual streamlined communication ‘channels’ on the main dashboard. These channels are customized and named with a hashtag to provide a forum in which participants can take part in discussions, tag and message each other, share documents, hold voice or video calls, share screens, and so forth in real-time or in an asynchronous format [10]. This allows for a knowledge-sharing environment focused on a larger series of specific projects, topics, or goals where information can be stored and collaborated on [11]. All files and messages posted in a group’s channels are searchable.

Slack has been successfully used by collaborative groups in a variety of settings to accomplish different goals within healthcare and healthcare education, including communicating with students in undergraduate medical education [12], for individualized small group graduate medical education instruction [13], for scientific conference discussions [14], to design programs for clinician wellness [15], and to coordinate institutional research [16]. Users of Slack report satisfaction with the platform, with nearly 80% reporting that Slack enriched their team culture and 88.6% feeling more connected to their teams [17].

Mobile Instant Messaging (MIM) platforms such as Slack and WhatsApp have been shown to offer a solution for bridging geographical and social divides to enhance learning, knowledge sharing, and collaborative problem solving in global health work across a range of low-income countries [18]. These types of digital tools offer convenience, ease of use, networked communication, and low costs platforms. While Pimmer, Lee, and Mwaikambo [18] included a small representative sample of global health practitioners, especially those from LMICs, their use of a questionnaire design did not allow for follow-up or clarifying questions and it was not focused on the unique needs of women leaders in global health.

### Emerging Women Leaders in Global Health (EDGE) Slack Network

The Johns Hopkins Center for Global Health runs an Emerging Women Leaders in Global Health (EDGE) program. This is an informal, heterogeneous network of women from across the globe that is actively growing. The program includes a leadership course, seminar series, networking events, and an active Slack group [19].

Participants from the course, seminar series, and networking events are invited to join the Slack group, which is the hub for this network. At the time of this study, the Slack group had 650 members and counting with 60–80 active (meaning they posted a message or read at least one channel or direct message) members on average in any given week. Some examples of active topic channels in the Slack group include #mentorship, #networking, #doctoralstudents, #failures-and-breakdowns, #in-search-of-job, #in-search-of-talent, #leadership-resources, #medicalstudents, #mother-scholars, and #strategies.

One goal of the EDGE program is to foster meaningful connections that lead to impactful experiences such as collaborations, resource sharing, mentorship, job opportunities, and leadership progression. From program feedback and interactions with program participants at our events, we have heard and read anecdotes that suggest real and meaningful connections are being made in the network. The objective of this study is to document tangible examples of positive experiences, connections, or collaborations resulting from connecting with other participants in the Slack network using case-studies developed from semi-structured interviews.

In this paper we present the process and results of semi-structured interviews that were conducted to capture qualitative descriptions of women’s involvement with the EDGE Slack network. Ultimately, we aimed to evaluate this network to understand how to better build, model, and scale advantageous networks of women leaders in global health moving forward. Based on our findings, we propose tangible avenues to further support current and developing women leaders in the field of global health.

## Materials and Methods

### Study Design

This study utilized a qualitative case study design to provide detailed descriptions of specific examples of impactful experiences that resulted from participating in the EDGE Slack network. We conducted semi-structured qualitative interviews with self-selecting participants from the network who volunteered to share their experiences that resulted from connecting with fellow Slack network members.

The study design and materials were approved by the Johns Hopkins Institutional Review Board.

### Participants

#### Recruitment

A purposeful sampling approach was used to identify participants who might give the most comprehensive and knowledgeable information about using the Slack network [20]. We recruited a convenience sample of participants to be interviewed for our case studies by sending out a call to participate to all members of the Slack network via the regular announcement listserv, EDGE Slack weekly newsletter, and a post on the Slack workspace. This process aimed to ensure that all participants received the same information and access to the call announcement.

We invited any individual participating in the Slack network who wished to share their story and positive experiences with participating in the network to be interviewed. Any participants who were open to voluntarily sharing tangible network stories were invited to participate. These anecdotes could include positive learning experiences, exposure to research, growing networking opportunities, interactions with colleagues in their field, learning opportunities and increasing new skills, valuable feedback about research, teaching, and so on, attracting mentors and/or becoming a mentor, sharing advice about opportunities for building one’s current career or future, job leads and/or opportunities, and any other experience that allowed the participant to personally gain from this network.

#### Sample

Seven women responded to the call for interviewees and participated in an interview. Out of these, six subjects (n = 6) were included in our final sample, as one interview (case 5) was not recorded due to technical issues. The participants ranged in age from 28 to 48 years old with a mean age of 35.2 years and were all women. The participants came from Africa and North America, with two participants from Nigeria, one Indian/American expatriate living in Canada, one Indian expatriate living in the United States, and two Americans.

### Data Collection Methods

Semi-structured in-depth interviews were conducted with each participant via Zoom with two interviewers present. Participants were contacted if they responded to the call expressing interest in participating to arrange a time for an interview. Participants were interviewed via Zoom at the agreed upon scheduled time. Zoom is a cloud platform for video audio conferencing and recording; only the audio option in Zoom was used to record and transcribe the interviews.

Upon the interviewer and participant meeting in the Zoom meeting room, the interviewer first read a consent statement and obtained verbal consent from the participants before beginning the interview. The interviewers then used the semi-structured interview guide described below to ask open-ended questions that allowed the participants to respond freely. Participants were free to refuse to answer any question and could choose to skip the question or stop the interview entirely. The interviews lasted between 18 and 40 minutes in duration.

#### Semi-Structured Interview Guide

A flexible interview guide (Appendix A) was created to present a consistent protocol guiding how the interviewer conducted the semi-structured interviews.

### Qualitative Data Analysis

The interviews were transcribed and then reviewed for key points using an outline for thematic analysis to derive short vignettes of each interview. The demographic variables were analyzed using descriptive statistics. We sent each participant a document with their case narratives and requested confirmations to ensure the accuracy of our summaries. We also asked the participants for their personal pronouns and she/her/hers pronouns were preferred by all participants.

## Results

### Case 1: Connecting to Expand NGO Work

Participant 1 is a 48-year-old who works at a university in Zaria, Nigeria. She has been in academic medicine for the last 14 years and is now considering a switch to a non-academic sector. She also started a nongovernmental organization (NGO) to empower women to make informed health decisions for themselves and their children. She first learned about the EDGE program at the Consortium of Universities for Global Health (CUGH) conference where the Center for Global Health hosted a virtual satellite session. She then began attending the EDGE seminars and subsequently joined the Slack workspace to support her navigation into a new career path outside of the academic sector.

Two tangible benefits that came from her participation in the Slack network are connections she made with fellow network members that may lead to future collaborations and authorship opportunities. Upon introducing herself in the Slack workspace, the first connection was with a first responder based in the UK who contacted Participant 1. They met virtually for 30-40 minutes to discuss the possibility and logistics of conducting a training for healthcare workers on first aid and basic resuscitation. The second connection who reached out to Participant 1 has a background in neurology and has been involved in rolling out neurological awareness programs in developing countries. In their virtual meeting, they discussed ways to improve care for epilepsy and other neurological conditions. Participant 1 found this extremely meaningful, as there are stigmatizing beliefs and superstitions around neurological conditions in rural areas of Nigeria, and creating training and awareness around these issues is important to the mission of her work. Participant 1 has hope that each of these connections may lead to future collaborations.

I think the [Slack network] gave me an opportunity to connect with amazing ladies [and] learn a lot by participating; so I think it’s a very good arena for being able to contribute to women in global health.

In addition to connecting with potential collaborators for her NGO work, Participant 1 gained authorship opportunities on several papers by connecting with other members of the Slack network to work together on some collaborative projects. This collaboration has led to more meaningful and deeper professional connections for her as well as a number of publications. As a researcher from a low-resource setting, she has benefitted from using online platforms for participation, networking, and collaboration opportunities that would not otherwise be possible due to location differences and costs and logistics of travel.

### Case 2: Connecting to Stay Up-to-Date in the Field of Global Health

Participant 2 is a self-identified South Asian 38-year-old woman living in Toronto, Ontario, Canada. She works as a program associate at a Canadian funding organization that supports teams in LMICs and Canada, and focuses on their Maternal and Newborn Health portfolio. She joined the EDGE Slack network after receiving an email that was distributed to Hopkins MPH alumni introducing the Slack group in late 2020. She initially joined the group to build her network, as she was ending a contract and looking for a job at the time. She wanted to connect with other Hopkins alumni based in Toronto as her Hopkins MPH program was virtual and she did not have the chance to build these types of connections during her studies.

She landed a job (unrelated to her participation in the network), and now uses the network to help stay up-to-date with all that is happening in the global health field, finding it valuable to hear about other people’s work through the connections she has made in the Slack network.

So it’s nice when I have a question, to just plug it into this network. And also make connections with people who are at different organizations, because there are a lot of folks within this network that are not necessarily associated with Hopkins. [It is] a diverse community where you could just ask a question and you would probably get a lead that will lead to something else.

Since she works for a funding organization, she also utilizes the Slack network as a platform to recruit innovators to receive funding from the organization where she works. As a result she has made a connection with someone whom her organization may offer funding to in the future. At the time of Participant 2’s interview, she and her organization were in ongoing conversations with the Slack contact whose team is based in India and they were considering the possibility of formally inviting the contact to apply for funding from their organization in the near future.

Personally, she has also benefited from the network because it allows her to feel like part of a more diverse community, with ties to the Hopkins community and members from other institutes and organizations around the world. She also appreciates being a part of a group of women in a similar field. While searching for a job at the height of the COVID pandemic, it was challenging to feel isolated during this process and hearing other’s experiences helped her feel like she was not alone.

### Case 3: Connecting to Develop a Capstone Project

Participant 3 is a 34-year-old MPH student (concentration in global health) working as a Research Associate with the Department of Global Health at the University of Cincinnati College of Medicine in the United States and is interested in oral health. She identifies as Asian with an Indian background. She was invited by a classmate in her MPH program and joined the Slack network in Fall 2021.

Since joining she has been active on the Slack network, browsing the networking and career transition channels. She accesses the Slack group at least once each day, sometimes more frequently. This has led to her establishing several connections that are proving valuable for her MPH capstone research.

I posted my background [in the Slack channel] and immediately [name redacted] responded and I think it was, like, within minutes, and I was impressed. I was like, wow! I just posted something!

Her first connection put her in touch with another connection within the oral health community where she connected with [name redacted] who is working on improving oral health of Latin Americans. After connecting with him via LinkedIn, they have had several Zoom calls discussing issues relevant to her MPH capstone project.

Then, as a result of a post on Slack, she learned about an oral health seminar organized by the CUGH. Her attendance at the seminar led her to make a connection with one of the panelists who was from Harvard. This connection provided a further referral to an oral health interaction group where she was able to later meet with the director of the group. Overall, she has found the connections she made through the Slack network to be very useful, helping her to find additional literature relevant to her capstone project and facilitating discussions with a doctor who is working on a similar oral health project in Tanzania.

This all happened so quickly, like, within the first week, and I was like, okay, I don’t know why I did not do this before… I just didn’t know about it. Yeah, forming those connections is really helpful… And then when I made these connections, I was like, oh my God, people should know about [this network], because you really get to connect.

### Case 4: Connecting for Doctoral Program Applications

Participant 4 is a 28-year-old, self-identified Caucasian woman residing in Toronto, Canada. She currently works as a clinical research project assistant in the Center for Global Child Health at The Hospital for Sick Children. Her research focuses on maternal and child health in the developing world. She joined the Slack network in Fall 2020 after attending an EDGE seminar after it was shared with her by her institution.

Engaging in the Slack network has been critical in Participant 4’s career development while applying to doctoral programs. Specifically, the network was beneficial in connecting Participant 4 with potential mentors. Before engaging with the Slack network, she tried to contact certain professors and mentors in doctoral programs she wanted to apply to without any responses. But then she reached out to the Slack workspace for assistance in connecting with these professors, and a Slack member was able to send an email to a potential professor, cc’ing Participant 4, and the professors responded within 24 hours. As a result of reaching out for help on Slack, she received connections with professors from both Johns Hopkins and UCSF. Subsequently, she submitted applications to doctoral programs with the professors as potential mentors and was waiting on the outcome at the time of the interview.

I benefited because [name redacted] connected me with the two different people I was hoping to talk to, and they immediately responded after that, so that was awesome…I’ve submitted my applications with both of those individuals [as potential mentors], and fingers crossed that I get accepted.

### Case 5: Connecting to Serve in a Mentorship Capacity

Participant 6 is a 35-year-old professional living in Towson, Maryland. She is a third-year doctoral candidate at Johns Hopkins Bloomberg School of Public Health. Ethnically, she identifies as Caucasian. Her research interests include maternal and neonatal health with a focus on evaluating and understanding the complexities of maternal and perinatal death surveillance in crisis-affected contexts. She became involved with the Slack network after attending the EDGE seminars in Summer 2020. She has been a part of the network since its initiation in 2020 and has attended most of the events organized by the program.

As a result of her participation on Slack and in the networking session, Participant 6 has benefited by making several new connections, mostly with undergraduate and graduate students in a mentorship capacity. Following meeting new connections at virtual EDGE networking events, students would reach out seeking advice on topics such as how to apply for a doctoral program, and she has had many conversations on Slack and one-one meetings on Zoom to connect and share advice. Participant 6 values the role of the Slack network in bringing together female professionals and researchers from the field of global health. Having been mentored by various key female leaders, Participant 6 is very grateful for the experiences that she has been through and is keen on being a contributor to this network by using it as a platform to share opportunities with others. As a researcher at Johns Hopkins, she receives many announcements about jobs and other opportunities and the network is a means of sharing those opportunities with a diverse group of female professionals and researchers.

I like [the Slack network] because it’s a good opportunity to meet people in our field. It’s nice. I feel privileged because I’ve had really great female mentors outside of Hopkins and, you know, throughout my career. I joined because I feel like I would love for everyone else to have that same opportunity. I think that very rarely do those who are not based in the US or within a prestigious bubble like Hopkins get these opportunities to network with women who are looking for similar things, who have similar goals and aspirations. So I think that is the most valuable thing.

### Case 6: Connecting to Invite Participation in a Mentorship Program

Participant 7 is a 28-year-old female who self-identifies as African, currently living in Abuja, Nigeria. She works in a humanitarian organization that provides aid focused on nutrition and food security and livelihood programs in the northeastern part of Nigeria, to assist in the humanitarian crisis there. Participant 7 also volunteers for an organization that helps young early career professionals in public health to navigate their journey in global health. She joined the EDGE Slack network in 2020 after receiving a link to the Slack group from a source she cannot recall.

She checks in with the Slack network once or twice per week and has used the Slack network to spread knowledge about the mentorship NGO where she volunteers. They work to connect students and young professionals with experts in global health, so she has posted global health mentorship opportunities in the Slack network and has successfully recruited volunteers. Although she is not sure exactly how many mentors and students applied as a result of seeing the Slack post, she has recruited at least one mentor who has provided mentorship and career guidance to two or three young professionals or students in global health. She would like to continue to use Slack to recruit additional mentors for the program.

[A Slack member] applied and she was selected as a mentor. I believe she has about two to three young professionals or students in global health that she’s providing mentorship assistance [and] guidance in career [decisions to] and [this type of mentorship is meeting] all the objectives we are seeking to track.

Participant 7 has also benefited from making some connections that assisted her with her graduate school application process. She connected with another student on Slack and with an individual from Nigeria at one of the program’s speed networking events who provided guidance on her applications. This individual from the networking event was an alumni of one of the universities the participant applied to and encouraged her work. She likes how the Slack network connects people from all over the globe that might not otherwise come into contact with each other. She has been exposed to lots of job opportunities, and, although she has not applied to these jobs, she has shared and reposted the opportunities as well as referred others to join the Slack network.

## Discussion

Advances in technology and global circumstances have contributed to an evolution in the way people communicate and exchange knowledge. Digital collaboration utilizing online tools such as the Slack network expands the reach of networking, collaborating, and mentoring opportunities for women seeking to advance their careers as leaders in global health. Digital platforms make participation independent from physical location to allow a larger and more diverse composition of members compared to face-to-face groups. This is especially important for increasing equality in access and overcoming barriers such as cost of travel, inability to take time away from work and family responsibilities, and obtaining visas. Digital tools also facilitate greater collaboration and interaction when in-person meetings are not feasible due to global issues like the COVID-19 pandemic.

Slack provides an organized digital environment that promotes connection, collaboration, and belongingness. It can connect women across a broad range of academic and career interests and geographic locations. It also allows for growth and evolution of interest areas as membership grows. Members in the EDGE Slack group reported that the network has successfully brought together different aspects of learning and networking, such as promoting women in the workplace and supporting them in climbing the leadership ladder.

While theoretically, an accessible and inclusive digital environment is exciting, it is worthwhile to also consider some of the challenges that come with engaging virtually. Globally, there is still a substantial digital divide that presents participation equity limitations along gendered, racial, and economic lines. Only 45% of people in developing countries have internet connection and in the least developed countries only 20% are connected to the internet [21]. Women generally have less access than men, and the speed and reliability of internet services vary greatly, with slower connection in less developed locations [21]. Those less likely to own a mobile phone or personal computer would not be able to participate as fully, or at all, in a program such as a Slack network.

Additionally, online social networks have been a source of misinformation, especially in the past few years during the COVID-19 pandemic [22,23]. Due to the nature of virtual platforms, anonymity allows people to post almost anything without much personal consequence, even if it is false, harmful, or misleading. With a virtual Slack network, there is risk that users join the platform and present themselves falsely or spread misinformation in the network. It is important for those managing the social networks to take measures to mitigate risk such as assigning network moderators, screening new group members as they join to try to gauge genuine interest, and removing users who abuse the platform.

### Best Practices for Utilizing Slack

#### Utilize more means for spreading awareness and visibility of the network

While the most common ways that interviewees learned about the Slack group were via an email or seminar offered by the EDGE program and via word of mouth from colleagues or classmates, participants encouraged the use of various means for spreading awareness and increasing the visibility of the Slack network. Participants felt that members benefit most from a diverse membership base extending beyond Johns Hopkins to various institutions and regions but also warned of not getting too large that the network loses its personal touch and ease of interaction.

Since the network is most valuable when it encompasses a diverse membership base, several participants encouraged recruiting members from a wide range of backgrounds, institutions, and regions to help with more active discussions, networking, mentorship, and collaborative opportunities. According to several of the participants, they learned about and/or shared the network with colleagues via word of mouth.

So I did not know about this network until I was told, [despite] being in the US. So it’s just that people don’t know, and then I told another friend. And I asked her to join…

Participants particularly felt that reaching out to other centers and organizations to promote the Slack workspace to individuals outside of the network would help to expand the reach and promote the addition of more members from all over the globe. One way to accomplish more word-of-mouth recruitment could be to have ambassadors or representatives at universities outside of Johns Hopkins that could spread the word about the existence of the network and increase a diverse membership base.

[I think that] having ambassadors to connect and spread the word [would help get more people to join from other areas of the world]…I’m glad I joined, and I would like to take it forward in some way…

Other participants first learned about the Slack network by attending one of the EDGE seminars. Since these seminars increase the visibility of the Slack group amongst women who are actively engaged in learning more about various aspects of global health, one participant suggested expanding the speaker base of those invited to give seminars. She recommended investigating if there are some audiences who have not yet been targeted or some areas of public health that have not been covered to share many different types of global health work and recruit those with diverse interests and backgrounds into the Slack group. Other participants reported first learning about the program and Slack group while attending an outside conference hosted by CUGH. Advertising the opportunity to join the Slack network at the end of conference and meeting presentations in different specialty fields of public health globally was suggested. One participant noted that giving people the opportunity to sign up right at the end of a meeting could help encourage uptake since people tend to pay more attention when a meeting is coming to an end.

And towards the end [of the meeting], you usually pay attention, like ‘Oh, if I have a question where am I going to ask or where I’m going to get [connected to this] network.’ So if we could contact these smaller meetings and communicate [with them to] see if this Slack group can be shown at every presentation, towards the end [this could help increase visibility of the network].

Other participants felt that posts and advertisements on social media platforms like Instagram could help to expand awareness about the Slack group.

### Design the network for internal motivation

One of the strengths of digital social networking tools like Slack for applications across many disciplines is that they provide a common platform where professionals can learn, discuss, or share resources with colleagues. This allows for the formation of a professional support network, collaboration to solve problems and generate solutions, and acceleration of the translation of evidence-based research into daily practice [24]. Members participate in such a digital platform to engage in opportunities to share knowledge beyond institutional and geographical barriers as well as give and receive support for personal and professional development.

The content and architecture of an online community platform like Slack is important for attracting and retaining participating members. This type of virtual community is characterized by voluntary participation and therefore grows, thrives, and continues to exist as long as ongoing participation of the community provides value to the members [24]. People want their interactions and online experiences to matter and feel relevant to them. Including adequate channels that cover a range of members’ interests with consistent engagement is important so that those who post receive acknowledgement and feedback. This helps to foster the emergence of trust in the platform, community, and relationships [25].

Online communities like the Slack network offer a unique strength in that they can provide new ways to reinforce internal motivation. For example, receiving interaction, reply posts, and positive feedback in the form of available emoji reactions when making a post reinforces an author’s internal motivation and encourages other members to pay attention to the thread, as the entire community can see who is engaging and following conversations [25]. In such an online community, a social environment characterized by collaboration and trust allows members to develop positive motivation and favorable sentiments toward each other that increase the willingness to actively participate [26].

One of the technical strengths of Slack is that it allows for an easily evolving platform that can accommodate a broad range of interests via the creation of new channels. This can provide opportunities for women across a range of subspecialities, work environments, and research interests to find a community of women like them as well as diversify their connections beyond their geographic and academic focus. It also allows the community to grow and evolve by adding new channels as the need or interest arises. This is especially relevant during times like the COVID pandemic, where new situations and current events are rapidly evolving and impacting public health at a global level. One participant suggested that the Slack network is an ideal platform to share an understanding of what is going on in the public health space and to discuss current events. For example, learning about what people are creatively doing to adapt to the circumstances created by the COVID pandemic and sharing lessons that will translate to other cultures and societies. She shared that this could help people get new ideas and expand their understanding of what is happening and is considered important in different regions and areas of global health.

Overall, participants we interviewed were utilizing Slack for a wide variety of purposes ranging from searching for or sharing job opportunities, finding mentorship partnerships, recruiting projects to fund, facilitating graduate school applications, developing programming and outreach ideas, and more. One participant also suggested being sure to provide additional value for those in more senior phases of their career and opportunities for women as they move up in their careers in addition to supporting women early on in their careers or who are looking to make a career change.

So I feel like there is a balance between folks that are in the later stages in their career, who may have opportunities to share, and then there’s the other side where there’s folks looking for opportunities. I feel like most times networks like this are usually skewed towards one direction and don’t benefit [you] if you’re on the other side of that spectrum. Maybe trying to think about what that balance would be [in the Slack network] instead of focusing primarily on [finding] careers and [expanding content around] what happens as you move up in your career.

Participants reported that they really value the opportunity to hear about other people’s work through the connections they are making in the Slack network. To them, Slack provides a benefit over in-person opportunities since it allows them to feel like a part of a diverse community of people they may not have otherwise had the chance to meet and interact with within their local workplace or academic setting. In particular, they highlighted the usefulness of having one place to be able to post questions or opportunities and reach a wide audience of people from different organizations, career paths, and subspecialties. Communities where members are more emotionally involved increase the desire for new members to join and actively participate.

And then when I made these connections, I was like, oh my God, people should know about it, because you really get to connect, people have your speciality here.

Studies suggest that such a virtual community that allows members to actively interact and discuss topics in a specific community leads to similar levels of emotional attachment to the virtual site as if they were in a physical place [27]. While other digital tools like LinkedIn allow for professional networking via direct messaging, Slack is unique as a learning and networking platform because it allows for the inclusion of a broad range of different topics via the creation of unique channels that allow women at different stages of their career to feel connected and included.

I would say that Slack is unique because it creates different channels to address different things; like if you want to network around doctoral programs there are platforms or channels [for that], and you can just zoom in and enter [that channel] and you see a specific request or areas of interest addressed.

### Encourage consistent engagement

Research has shown that communities where there is consistent interaction among members, attract more members and result in increases in the knowledge sharing activity which continues a cycle of greater participation [24]. In our study, the levels of interaction on Slack varied among participants between checking the app and interacting with the network once or twice per week and being more actively engaged. Despite this variation, participants highlighted the importance of posting regularly on Slack to strengthen the network and increase engagement. They described various ways that can be adopted by both network organizers, to create a more consistent content, and network members, to increase their engagement.

Some suggestions for network organizers included regularly posting questions and making more opportunities available for members to contribute to discussions. Recommendations for increasing consistent engagement by members include regularly checking notifications particularly those that are relevant to their posts and questions, revisiting their previous posts as they might become updated, subscribing to the newsletter, and directly messaging members with whom they share specific interests.

Networking takes time and it’s something you also prepare and plan for, so I think it’s maybe one [tip] that seeps out is to be intentional in seeking out people. Really be intentional. Search their name - so beyond being on Slack check them out on LinkedIn. Check out their work. What are they doing? Is it related to what you’re doing? Are you interested? And if you go to form an interest, then you can go ahead and send a message, add a note.

This is aligned with the emphasis on active participation described by Antonacci et al. (2017), which results in acquiring experiences and stimulates the development of knowledge in a social context and the development of skills and expertise [24]. Other benefits at the network level include keeping the community alive and broadening participation [24].

I really like how you constantly toss some questions in there and keep it [Slack network] active. I think that’s really good because it would be easy to just forget about it, otherwise.

One important window of opportunity in particular that members should seize in order to enhance their engagement is to add a personal touch at the time of joining the network by sharing relevant insights about themselves and their interests in an introduction post. This timing is favorable because introduction posts with personal touches help start conversations according to participants.

It’s always nice that somebody on the channel is encouraging people who are joining the group for the first time to provide introductions…Sometimes it is nice to put in some personal things in there as well.

Participants indicated the importance of members personalizing their posts including introductions and being more intentional and purposeful in their approach to engaging with the network (e.g., learning about people’s work before messaging them). Several participants shared that adding a personal touch to one’s own posts helps members get the most out of the Slack network.

It’s nice to know somebody a little bit more than what they do and what they aim to be in their careers. Yeah so maybe adding some personal stuff might be like a little bit of a conversation starter.

Our findings are consistent with the literature on the relationship between personalization and community engagement. [28,29] Studies have found that personalized communication encourages members to access the network’s website and enhances the level of interaction and satisfaction toward the network [29]. Personalization helps in identifying roles of individuals within the network (e.g., moderator role) which in turn maintains engagement [24].

### Send weekly emails

Slack has been successfully utilized as a networking platform in diverse health care settings [30]. It has the potential to lead to more effective networking in global health, particularly amongst women, as women may not have the opportunity to use other traditional networking opportunities available for men due to fulfilling additional roles outside of work and study.

Several of the participants interviewed felt that the weekly email newsletter sent out to registered members every Tuesday is a key strategy for making the Slack network successful. The weekly email update contains a welcome message, highlights from the Slack workspace, notifications about any upcoming program events, a discussion of the week, an introduction to the various channels and how to use them, and a Slack member spotlight that introduces a member of the Slack community to the entire group in more detail. This Slack member spotlight introduces a member by name and highlights their qualifications, affiliations, interests, future prospects and aspirations, and main professional goals.

The concept of this weekly email newsletter was sparked by a response to feedback given by members of the Slack network. Participants provided feedback that they were unsure exactly how to use Slack and that they didn’t think about utilizing Slack even though they felt it had value. Therefore, the weekly email newsletter was developed to provide a recap for new members about how to access and use Slack, introduce information about specific types of channels available for interaction, and prompt members to think about the network and log in regularly to participate.

According to the interviewees in our study, the newsletter is meeting these objectives. Interviewed participants reported that the weekly emails serve as the best reminder to log into Slack and help them optimize engagement with the network. The newsletter complements the role of the Slack network particularly for those who turn off notifications, are interested in receiving a summary of all opportunities, are not very comfortable with using Slack, or have internet connection problems. When asked how often she checks Slack, one participant responded:

I can say that I particularly like the every Tuesday mail that gets sent out. I find that very useful because, even if I don’t log in and look at the Slack network, I have an opportunity to see at a glance what’s going on and any other thing that catches my interest. I now log on and look in more detail about that particular event…so the most important aspect of the Slack network is the email that gets sent out every Tuesday. I find that particularly useful.

### Maintain accessibility for a global membership base

One of the key strengths of the EDGE Slack network that all the participants appreciated was its global nature and diverse membership. Participants applauded how the Slack network expands beyond the Johns Hopkins community and targets internationally based students and researchers to serve as a platform for women from less privileged backgrounds to connect with others who share similar interests and goals. In addition to expanding recruitment and advertising about the network globally, as mentioned previously, it is important to maintain accessibility within the network for a global membership base.

To make the network most accessible to all, one participant suggested expanding features for non-English speakers and for specific subgroups. This could be done by simple helpful means such as providing transcripts and subtitles and hosting events tailored to specific subgroups. Another participant suggested adding additional channels for subgroups and specific topics of interest where groups could work together on specific topics.

Another thing [that would add value to the network] would be having people who are interested in particular activities create a space for a project within the Slack network.

Evidence shows that even though market participation among women fell during the pandemic, their involvement in online learning proposals increased 23.4% in 2020 when compared to historical data [31]. So, in a sense, the global health crisis brought about a huge opportunity to narrow the gap in opportunities in terms of training, networking, and collaboration among women. Thanks to the pandemic, people are conditioned to be able to work and contact each other remotely and use online platforms for collaboration which has opened up avenues for expanded participation that can benefit a global audience.

Participants highlighted the relevance of the Slack community for making participation more feasible for professional women living around the globe. As one participant highlighted, as a researcher from a low-resource setting, attending major conferences and meetings in-person would have been very challenging given the cost and logistics involved in traveling overseas, registering for the event, and getting visa technicalities sorted out, but online sessions and platforms gave her the opportunity to participate from her home.

In particular, participants reported valuing how the EDGE Slack network offers the capability for collaborations between people spread geographically far apart. One participant highlighted how it can be used to enable health equity globally by bringing together researchers and practitioners from the Global South and the Global North to work on particular projects together and to share data and insights. In her words,

I think the Slack network has an opportunity to be able to position itself as a go to for information on [collaborations- both North and South and South and South collaborations].

Countries in the Global South still struggle to provide training, networking and development to global health researchers [32], so digital tools like Slack can help reduce the gap in opportunities for women working in peripheral countries compared to women working in central economies [33].

### Strengths and limitations

This study had some limitations including a small sample size and the fact that the majority of the respondents were from the Global North. This limits the generalizability of the findings to broader global settings. Further, this research represents a convenience sample since the respondents were recruited from the EDGE Slack network. Therefore, this is not representative of all women working in global health. In addition, given the nature of the case study design there may inherently be subjectivity bias introduced by the interviewers.

Additionally, because the recruitment and interviews focused specifically on positive outcomes of participating in the Slack network in this initial evaluation, the case studies themselves do not include any potential negative outcomes of participating. However, constructive feedback was provided by the participants and the suggestions were considered for this manuscript. As the program matures, a more comprehensive evaluation in which outcomes are assessed with a more neutral methodology should be conducted.

There were also several strengths of this study. This is among one of the first studies that has examined the role of digital tools, specifically Slack, in connecting women in global health for job searching, mentorship opportunities, graduate school applications, and other opportunities that can help them advance their opportunities and leadership potential. In addition, the narratives provided richness, gathering detailed descriptions of diverse experiences from the participants. Moreover, given the breadth and depth of the study, this study has achieved thematic saturation.

## Conclusion

### Overall evaluation of the Slack network

Overall, the findings of this study indicate that Slack is a highly beneficial tool for women in global health to use for facilitating job searches, mentoring opportunities, promoting project collaborations, and proposing programming and outreach ideas in a remote working environment. To facilitate the use of Slack in a way that best supports and engages women in global health, we found it is important to spread awareness and ensure visibility of the network to recruit and maintain members, design the network in a way that inspires internal motivation, encourage consistent and meaningful engagement, send weekly emails, and maintain accessibility for a global membership base.

While the era of digital means of networking was enhanced during the COVID-19 pandemic, Slack offers a sustainable tool for women beyond the pandemic to continue advancing their academic and leadership interests in global health, particularly for individuals who may not be able to participate in on-site events. In fact, digital tools such as Slack can help to close the gap in global health leadership disparities by increasing the opportunities for participants from low- and-middle-income countries, who may not have previously participated due to lack of resources or access, to engage in the same networking and leadership opportunities as individuals from high-income countries, though access to internet is still a barrier to consider. Moving fully back to in-person meetings or conferences without a hybrid option to include virtual content once pandemic restrictions are eased would be a great disservice. This would disproportionately impact women especially those who are disabled and individuals from low-and middle-income countries who encounter a myriad of barriers to attending in-person events including astronomical costs of attending conferences, navigating travel logistics such as procuring a visa, and being unable to take time away from other responsibilities such as caregiving. Failing to provide greater accessibility to opportunities via digital tools would further alienate existing and new relationships and expand imbalances in power between the global north and south which are rooted in harmful neocolonialistic approaches. Subsequently, this would disrupt the efforts and goals of decolonizing global health. Therefore, it remains critical to continue to build, advance, and scale advantageous networks like Slack to promote equity and accessibility among women leaders in the global north and south into the post-pandemic world and to evaluate how to make such digital tools most sustainable as the world evolves.

## Appendix A

**Semi- Structured Interview Questions for Johns Hopkins Emerging Women Leaders in Global Health Program Slack Case Study Participants**

1. Introduction:
  1. What is your name?
  2. What is the location where you work and live currently?
  3. What race, ethnicity and/or cultural background(s) do you identify with?
  4. What is your age?
  5. What is your current work?
2. How did you get involved with the Johns Hopkins Women in Global Health network?
  1. Probe: When did you join the network?
  2. Probe: What events have you participated in through the network?
  3. Probe: How often do you use the network ie Slack?
3. How did your participation in the network benefit you?
  1. Probe: What is the nature of the benefit you experienced? (This could include meaningful connections, collaborations, projects, leadership opportunities, job opportunities, hiring opportunities, mentorship, future opportunities, or any other positive result of participating in the network.)
  2. What was the timeline of this beneficial experience?
  3. Probe: Who initiated the connection?
  4. Probe: What is the current status of the connection/collaboration?
4. What role did the network play in facilitating the benefit you described?
5. What do you think were the key ‘ingredients’ for this positive connection/outcome to happen?
  1. Probe: What was special or unique about this particular network that led you to this kind of benefit?
6. How can others in this network begin to experience the benefits of being in this kind of group? What tips do you have for them?
7. What are any suggestions you may have for how we might further strengthen the network to foster more connections/outcomes?
  1. Probe: Do you have suggestions for improving the **quantity** of connections?
  2. Probe: Do you have suggestions for improving the **quality** of connections?
